# Supplementary material for: The acceptability of intimate partner violence screening and response among refugee women accessing Australian resettlement services
Source: PLoS One. 2024 Dec 18;19(12):e0315061. doi: 10.1371/journal.pone.0315061 (PMC11654948; doi:10.1371/journal.pone.0315061)
Supplement: Attachment — Submitted filename: PONE-D-24-04846 comments.docx [file pone.0315061.s001.docx]

This was a very well-written and timely study that will be extremely valuable globally! I really appreciate the care that the authors put into the study. To be honest, I found little to squabble with, but mostly have comments that I hope can strengthen what is already a strong piece of work. Thank you for doing this work and reporting on it.

**Overall Comments:**

There is an unstated assumption in this article that the primary purpose of screening women for IPV is to get a disclosure. But we know that survivors experience numerous barriers to disclosure and may not disclose when asked. Screening certainly provides survivors the opportunity to disclose their experiences and receive support and referrals to services. But it also may have benefits for survivors beyond disclosure, particularly when done in a trauma-informed and empowerment-centered way. While an older article, Spangaro, Zwi, and Poulos (2011)’s qualitative work suggests that being asked and naming abuse was important. More recently, work by *Futures Without Violence* recommends universal education and empowerment, and while that may not include asking a direct question, still suggests that initiating conversations can be beneficial. My primary suggestion in saying this is to be clear about the goals of screening women in refugee settings – is it simply to encourage disclosure? This is the outcome the authors most frequently discuss, at the exclusion of other benefits that can come from broaching the subject.

**Specific Sections:**

Introduction:

Line 71: “Universal screening for IPV in health care settings is recommended…”

Methods:

Line 152: rather than a comma after “groups” there should be a colon (:)

Participant Recruitment (Pg 9): were women who went to the center multiple times over that time period asked multiple times to participate in the survey? Also, the authors refer to “Chinese” as a language, but later it sounded like it was Mandarin? Maybe this could be more specific.

Pg 10 line 202: To make it clear that participants were only contacted once by RAs, maybe start this with “When participants were contacted, they were provided further information…”

Line 207: I found the phrase “before commencing the survey with the opportunity to stop the survey at any time” really awkward. Maybe “The study’s safety and privacy protocol included checking with the participant that her situation was safe, private, and convenient, and letting her know that she could stop answering questions at any time before commencing with the survey questions.”

Pg 11 Line 209: the follow up period was three months – I just wonder if the authors have any data describing how long it often takes women between a disclosure of violence to when they take some type of action?

Line 213: how were the participants who disclosed violence given the shopping voucher if they were living with the person causing harm?

Results:

Pg 12/13, Participant Profile: Did women have to show visas to receive services at SETS? In other words, was it clear if any of the participants were in the country w/o authorization or legal status? It would be good to know how this could affect screening and response.

Pg 16, First or prior experiences of screening: For the 13 women who said that the SETS screening was the first time they disclosed, were they asked if it was the first time they had been asked? Can the authors explain why they removed the 27 participants who said they screened positive for IPV during the initial visit from the analysis of being screened by other services? It seems like useful information to know if they had ever been screened elsewhere, regardless of whether they disclosed.

Pg 18, line 336: this might be better located in the discussion. Also, I think the authors mean that women who remember being asked are more in favour, not women who were asked.

Pg 19: there is a typo in either Table 6 or in the text where referring to the number of respondents say “So women can get help/be kept safe.” The table has 159, the text says 156/314.

Pg 20 lines 383-386: are these from different women or from the same person? The formatting makes it hard to tell. The authors could consider referring to individual respondents as “Participant 1” if they do not feel comfortable using additional descriptors.

Pg 20-21, What would make a difference: This section is confusing as it is written, particularly the part on Pg 21 that tries to describe who rated which statements highly. I understood what the authors meant, but only after looking at the table closely.

Discussion:

This might be a place where my comments about the intention of screening always being disclosure could be addressed.

While I do not want to put undue weight on the responses of people who disapproved or would be uncomfortable with IPV screening, is it possible for the authors to provide a little interpretation about the reasons those few people gave against screening, and what that might suggest about the approach that refugee service providers take? I realize these were very few in number compared to those who support this practice, but providers often say that fear of offending patients (particularly related to cultural relativism) is one reason why they don’t want to talk about IPV, so acknowledging that while some participants felt uncomfortable with being asked, it was far from the majority. We need to move away from stereotypes about the overall acceptability of IPV among some communities.

Limitations: I do not understand how service improvement by SETS to better respond to clients experiencing IPV was a limitation of this study, looking at the acceptability of IPV screening in the setting. In terms of the research team offering assistance to the SETS sites, it would be useful to reflect on what that assistance was (presumably IPV training, support with asking difficult questions, etc.) and rather than seeing that as a limitation, reflect on it as a necessary part of a screening program for other refugee assistance settings.

Is an additional limitation that some participants in the survey did not actually receive the intervention, but cannot be separated from those who just don’t remember if they received the intervention? That seems worth reflecting on.

Tables

I will let the copy editors work with authors on formatting the tables. I don’t usually see shaded lines, and while I figured out what “IPV+ve” and “IPV-ve” mean, I don’t think it’s standard. It’s also nice when I see information about the population included in the title, even just something as simple as “all participants.”
