# Supplementary material for: The acceptability of intimate partner violence screening and response among refugee women accessing Australian resettlement services
Source: PLoS One. 2024 Dec 18;19(12):e0315061. doi: 10.1371/journal.pone.0315061 (PMC11654948; doi:10.1371/journal.pone.0315061)
Supplement: Attachment — Submitted filename: Response to Reviewers.docx [file pone.0315061.s002.docx]

15 October 2024

Michelle L. Munro-Kramer, PhD, CNM, FNP-BC, FAAN
Academic Editor
PLOS ONE

Dear Professor Munro-Kramer

The authors appreciate the encouraging feedback, and the many questions and proposed changes from the reviewers. These have been extremely valuable in improving the manuscript.

The table below provides a detailed response to all reviewer comments, with changes made as indicated in the table and in the tracked changes version of the manuscript.

Additionally, here are the responses to the Journal requirements:

1. Please ensure that your manuscript meets PLOS ONE's style requirements, including those for file naming.

Response: we have reviewed the style requirements and believe the manuscript to be compliant.

2. Please provide additional information regarding the considerations  made for the refugees included in this study. For instance, please discuss whether participants were able to opt out of the study and whether individuals who did not participate receive the same treatment offered to participants.

Response: all women participating in the study were explicitly informed as part of the information and consent process that they could withdraw from the intervention and could withdraw from participation in the survey at any time without question, and this would have no bearing on their continued receipt of service from the settlement service or any other organisation.

3. Thank you for stating the following financial disclosure: “This study was funded by the Australian Research Council (LP190101183) and SSI, a large non-government agency providing settlement services based in Australia.” Please state what role the funders took in the study.

Response: The Australian Research Council had no role in study design, data collection and analysis, decision to publish, or preparation of the manuscript. SSI had two chief investigators on the study governance committee who contributed ideas to the study’s implementation, data analysis and preparation of this manuscript, while at all times the University of Wollongong investigators retained full control of study design, implementation, data collection and analysis, decision to publish, and preparation of the manuscript.

4. We note that your Data Availability Statement is currently as follows: [All relevant data are within the manuscript and its Supporting Information files.] Please confirm at this time whether or not your submission contains all raw data required to replicate the results of your study.

Response: The authors confirm that all data required to replicate the study are contained within the manuscript.

5. Please review your reference list to ensure that it is complete and correct.

Response: the reference list has been reviewed and we believe it is compliant with PLOS ONE’s requirements.

Kind regards,
Nigel Spence (Corresponding author)

| **REVIEWER COMMENTS** | **RESPONSE TO REVIEWERS** |
| --- | --- |
| 1. Have the authors made all data underlying the findings in their manuscript fully available? | The data for this aspect of the study is provided as part of the manuscript. |
| **Reviewer 1** |  |
| 2. Some contextual information would be beneficial. For those who do not know Australian context, it would be good to provide more details about what the resettlement services do. In addition, the total number of resettlement service centers in the country and major cities so that we can judge the relative representativeness of the sample. How many visitors does a center receive per day or per year? How many (or what proportion) are refugee women? | Additional contextual data has been included on page 7 and pasted here to describe the overall scope of Australia’s humanitarian support program and the services provided. Some data such as the number of service outlets and number of visitors per day are not available.  *The Australian Government operates a permanent settlement program for refugees with the number of places determined annually. In 2016-17, the program had 13,750 places, increasing to 16,250 places in 2017-18 and 18,750 places in 2018-19 (9). Support for refugees is provided by government funded services delivered by community organisations across Australia, mainly in the major urban centres. The study was conducted with women accessing one of the principal support services, the Settlement Engagement and Transition Support service (SETS) that provides support for refugees from about 18 months to five years post-arrival (37). SETS is a follow up program to the intensive case-management support service provided to refugees for the first 18 months after arrival in Australia, the Humanitarian Support Program (HSP) (38).*  *Families and individuals are referred to a SETS provider with the referral including visa and other relevant information that confirms their eligibility to access the SETS service. Using the SETS service is voluntary. SETS provide a wide range of services including to access a range of individual and group supports including introduction to the legal, education and health systems; language courses; citizenship courses; assistance to obtain short- and long-term accommodation; assistance with English language skills, access to employment;, housing, family issues, transport;, civic participation, citizenship, legal and administrative systems ; personal and family issues (39, 40).* |
| 3. When explaining the ACTS tool on page 5, it is not clear how the scores are obtained. Are there four questions with four possible answers or more questions within each ACTS category? If four questions, does each question get a response 1-4 (or 0-4) and do these reflect severity or frequency of exposure to IPV? | The ACTS tool contains just four questions that are each rated on a frequency scale comprising: Never (0) Rarely (1) Sometimes (2) Frequently (3) and Very frequently (4). Total scores can range from 0 – 16 and giving an indication of the frequency of IPV experience. To explain this better in the manuscript, the text has been amended on page 5:  *The four-question tool, titled ACTS, asks women how often in the last 12 months a partner or former partner has made them* ***A****fraid,* ***C****ontrolled,* ***T****hreatened or* ***S****lapped/ physically hurt them, with responses scored on a frequency scale with options for Never (0), Rarely (1), Sometimes (2), Frequently (3) and Very frequently (4). A positive screen for IPV ranges from the threshold score of 1 to a maximum of 16 and provides an indication of the frequency of exposure to IPV.* |
| 4. The stated rationale behind the study seems to be that refugee women’s views and acceptability of IPV screening in resettlement service centers are not known. It is not clear what the expectations are from theory or prior evidence for women feeling differently about screening at resettlement service centers as opposed to health care centers. | The Introduction section of the manuscript highlights evidence from other research that: refugee women are reticent to use formal services, IPV screening has been done successfully in healthcare settings, IPV screening is untested in settlement services. This leads to the possibility that settlement services might be effective sites for IPV identification, warranting exploration. We have added a sentence on page 6 to make this more explicit.  *… screening of refugee women in settlement services is untested and refugee women’s views about the acceptability of IPV screening are unknown. Settlement services are frequently accessed by refugee women and have the potential to be suitable sites for IPV identification and response.* |
| 5. It is mentioned that IPV screening is typically done in relation to health care providers visits. There are some downsides to it, but it is not very clear what the advantages of IPV screening at the resettlement service centers would be. I am not sure what the advantage is apart from resolving language barriers. This should come out clearer. Please elaborate. IPV screening in resettlement service centers could also have downsides. | In the Introduction and Conclusion we make the point that women should be offered the opportunity to speak up in safe environments about experience of IPV – if they so choose. Additionally, as addressed in the previous point (above), refugee women may be wary about speaking to formal, mainstream services. Settlement services can offer another site (not the only site) for disclosure of IPV and have the advantage of language matching and cultural safety. This assumption is supported by the findings reported in the Results section, particularly on pages 20-21, and briefly in the Discussion section on p.23. We think the findings in the Results section point to the advantages of screening at settlement service sites. Possible downsides are addressed below. |
| 6. One potential downside could be that women might be afraid about talking about IPV at the same place they go to look for job (or seek information about jobs; this is not clear to me as I do not know the Australian context and the details are not given in the paper). If they fear that a future employer might discover something private about them such as IPV they may be reluctant to talk about it in resettlement centers. Similarly, they may fear visa or other paperwork being denied. I am not sure about how IPV is treated legally in Australia. Are there fines or sentences for perpetrators? If so, women may not be comfortable about IPV screening at resettlement service sites as they fear legal persecution or deportation for their partners. | IPV is a criminal offence under all Australian state and territory legislation with a range of penalties applying. There are many enablers and barriers to talking about IPV identified in our study that are reported separately (papers are under review). We have not been able to cover everything in this manuscript but agree with the reviewer’s broad comment and have added the following on page 23:  *… multiple barriers to speaking up about IPV for refugee women are identified (reported separately) including fear of retribution, concerns about the consequences of disclosure, not wanting to break up the family, economic insecurity, lack of knowledge about Australian laws and services, visa insecurity, and complex relationships with communities (55).*  Regarding the specific concern that women may fear their personal information being shared with employers or other services, settlement services in Australia are bound by a Settlement Services Privacy Policy as well as organisation-specific confidentiality policies that prohibit sharing of personal information without consent. |
| 7. Were the shopping vouchers for a specific shop? If one particular shop (or a chain of shops), why was it chosen? Could the shop choice deter participation? | The shopping vouchers were for one large chain of shops (Woolworths) with multiple outlets across the areas where the service sites were located, and further away. Women could choose a store convenient and safe for them. No concerns were reported by any of the women or service providers. |
| 8. It would be very useful to state in the methods section which eight enablers were used to code the qualitative responses. | Agreed. The eight categories have been added to the methods section on pages 10-11. |
| 9. It is difficult to understand the numbers on participation. It is stated that out of 375 women that agreed to take part, 321 were surveyed, 30 were unable to be contacted, 19 declined and 1 survey was not completed. The sum of 321+30+19+1 is 371. What about the remaining 4? Also, for each discrepancy between the recruited and surveyed women, please give a number (e.g. excluded and not asked the screening questions). | Thank you for identifying this discrepancy. There was a small error in the original manuscript regarding the number unable to be contacted and the number of surveys not completed. This discrepancy has been corrected in the manuscript on page 13:  *…Another 32 women were unable to be contacted, 19 declined and three surveys were not completed.* |
| 10. More precise sentence phrasing would be beneficial. For example, the last sentence in the first paragraph would benefit from clearly stating the comparison group for IPV affected refuge women (e.g. host or origin country population). It is also not clear how vulnerability of refugee women should be understood in the last paragraph on page 4 (is this vulnerability to IPV or broader vulnerability?). | The paragraph referred to (page 4) has been amended to improve clarity:  *Women from refugee and immigrant backgrounds who are settling in a new country are at greater risk of IPV than women born in the country of settlement*  The reference to vulnerability (now in the first paragraph on page 5) has been amended to clarify that it is vulnerability to IPV. |
| 11. On page 8, it is stated in the four main languages as well as English and three other community languages. Please check if there is duplication here as later on only four languages in total were mentioned. | This is a drafting error and has been corrected. The study tools were in English as well as the four languages most spoken by women visiting the sites - Arabic, Farsi, Chinese and Vietnamese. |
| 12. Change the indicator of affiliation from 2, 4, 9 and 10 to 1 as it appears that this is the same institution. | The indicator affiliations have been amended to show that multiple authors are affiliated to the first listed institution. |
| **Reviewer 2** |  |
| Overall Comments: |  |
| 13. This was a very well-written and timely study that will be extremely valuable globally! I really appreciate the care that the authors put into the study. To be honest, I found little to squabble with, but mostly have comments that I hope can strengthen what is already a strong piece of work. Thank you for doing this work and reporting on it. 14. There is an unstated assumption in this article that the primary purpose of screening women for IPV is to get a disclosure. But we know that survivors experience numerous barriers to disclosure and may not disclose when asked. Screening certainly provides survivors the opportunity to disclose their experiences and receive support and referrals to services. But it also may have benefits for survivors beyond disclosure, particularly when done in a trauma-informed and empowerment-centered way. While an older article, Spangaro, Zwi, and Poulos (2011)’s qualitative work suggests that being asked and naming abuse was important. More recently, work by Futures Without Violence recommends universal education and empowerment, and while that may not include asking a direct question, still suggests that initiating conversations can be beneficial. My primary suggestion in saying this is to be clear about the goals of screening women in refugee settings – is it simply to encourage disclosure? This is the outcome the authors most frequently discuss, at the exclusion of other benefits that can come from broaching the subject. | The authors appreciate the encouraging feedback and have found the comments to be very helpful in improving the manuscript.  Completely agree. It was not our intention to suggest disclosure is the only reason to undertake screening. Education, raising awareness about the issue (for staff/workers as well as clients), empowering women, affirming women’s rights to safety and creating opportunities to get help, are some of the other benefits of an IPV screening intervention. We have:   - made a small amendment on page 5:   *Asking women directly about experience of violence, raises awareness about the issue, affirms women’s right to safety, increases disclosure and creates opportunity for supportive intervention (25, 26).*   - added to the Discussion on page 24:   *Women’s responses in the study also point to the benefits of screening that extend beyond disclosure of IPV, with participants commenting on the value of creating opportunities for women to get help, education and awareness raising about IPV, affirming women’s rights to safety and empowering women who may feel isolated.* |
| **Specific Sections:** |  |
| Introduction: |  |
| 15. Line 71: “Universal screening for IPV in health care settings is recommended…” | This change has been made to what is now Line 72. |
| Methods: |  |
| 16. Line 152: rather than a comma after “groups” there should be a colon (:) | This change has been made to what is now Line 158. |
| 17. Participant Recruitment (Pg 9): were women who went to the center multiple times over that time period asked multiple times to participate in the survey? | Women visiting the centre multiple times were only invited once to participate in the survey. As there was one continuous member of the study recruitment team stationed at each site they could recognise if they had previously asked the woman who was visiting. On rare occasions when the recruitment team member could not remember, the woman was able to point out that she had already been asked. |
| 18. The authors refer to “Chinese” as a language, but later it sounded like it was Mandarin? Maybe this could be more specific. | The terms Mandarin and Chinese are used quite specifically in the manuscript, Mandarin being the spoken language – as spoken by women, staff and study team members - and Chinese (specifically ‘simplified Chinese’) being the written form of the language used in study tools. |
| 19. Pg 10 line 202: To make it clear that participants were only contacted once by RAs, maybe start this with “When participants were contacted, they were provided further information…” | This change has been made to what is now Line 208. |
| 20. Line 207: I found the phrase “before commencing the survey with the opportunity to stop the survey at any time” really awkward. Maybe “The study’s safety and privacy protocol included checking with the participant that her situation was safe, private, and convenient, and letting her know that she could stop answering questions at any time before commencing with the survey questions.” | Thank you, we agree this phrasing is better and has been used to replace what is now Line 212 |
| 21. Pg 11 Line 209: the follow up period was three months – I just wonder if the authors have any data describing how long it often takes women between a disclosure of violence to when they take some type of action? | Earlier research, including studies by some of the authors found that 3-4 months is a time frame long enough for women to take action following an IPV intervention while being soon enough for women to remember the intervention. This is why the time period was selected for the study being reported. However, more broadly, the time frames between experience of IPV, disclosure and taking action are found to vary widely, are influenced by many factors and are not always linear.  To address this in the manuscript the text has been amended on page 11 and additional references provided referencing relevant studies:  *The follow-up period of three months was selected as a time period found in other research to be sufficient for possible action by women following the intervention, and soon enough for most women to remember the intervention (49, 50), while recognising that women choose many different pathways and time frames for action (51, 52)* |
| 22. Line 213: how were the participants who disclosed violence given the shopping voucher if they were living with the person causing harm? | Women were given the option to have the voucher sent by SMS to their phone, or to their email or by post. The sending organisation was the University and did not identify anything about the voucher being related to an IPV study. The choice of how to receive the voucher and the generic nature of the information sent were deemed, in consultation with our women’s panel and caseworkers, to be a safe mode of delivery. Most women opted for receiving it via SMS to their phone. |
| Results: |  |
| 23. Pg 12/13, Participant Profile: Did women have to show visas to receive services at SETS? In other words, was it clear if any of the participants were in the country w/o authorization or legal status? It would be good to know how this could affect screening and response. | Women are referred to the SETS service by their initial support service provider (usually an HSP service). The referral includes their visa type and other information which confirms their eligibility to receive services at SETS. SETS services are not supposed to assist ineligible clients. To our knowledge there were no unauthorised refugees amongst the participants. We have added the following on page 7:  *Women are referred to a SETS provider with the referral including visa and other relevant information that confirms their eligibility to access the SETS service. Using the SETS service is voluntary.* |
| 24. Pg 16, First or prior experiences of screening: For the 13 women who said that the SETS screening was the first time they disclosed, were they asked if it was the first time they had been asked? Can the authors explain why they removed the 27 participants who said they screened positive for IPV during the initial visit from the analysis of being screened by other services? It seems like useful information to know if they had ever been screened elsewhere, regardless of whether they disclosed. | The 27 participants who said they screened positive for IPV during the initial visit were asked many questions, including whether this was their first disclosure, but were not asked if they had been screened elsewhere. We agree this would have been valuable information however there were a large number of questions asked of these participants, we were concerned not to burden or possibly traumatise women with the questions, and Ethics approval required that we reduce the questions asked of this group. |
| 25. Pg 18, line 336: this might be better located in the discussion. Also, I think the authors mean that women who remember being asked are more in favour, not women who were asked. | Agreed. This point is already in the discussion so we have deleted the sentence referred to be the reviewer and amended the sentence in the Discussion on page 23:  *It is also noteworthy that women who weren’t asked the questions or did not remember being asked, had lower agreement and higher disagreement than those who remembered being asked the questions, indicating that women are inclined to be more in favour when they have had direct experience of being asked.* |
| 26. Pg 19: there is a typo in either Table 6 or in the text where referring to the number of respondents say “So women can get help/be kept safe.” The table has 159, the text says 156/314. | Thank you. This has been amended in the text to 159/314. |
| 27. Pg 20 lines 383-386: are these from different women or from the same person? The formatting makes it hard to tell. The authors could consider referring to individual respondents as “Participant 1” if they do not feel comfortable using additional descriptors. | Every comment is from a different woman. The anonymised, individual descriptors have been added. |
| 28. Pg 20-21, What would make a difference: This section is confusing as it is written, particularly the part on Pg 21 that tries to describe who rated which statements highly. I understood what the authors meant, but only after looking at the table closely. | The text, now on page 22, has been amended to clarify meaning:  *The most highly ranked factors were ‘care shown by the worker’, ‘talking to someone in my own language’, ‘trust in the confidentiality/privacy of the service’ and ‘talking to a female worker’ (Table 7). These four factors were ranked highly by all women – those who disclosed IPV and those who didn’t. The women who disclosed IPV also identified ‘knowing and trusting the worker’, and ‘feeling confident the worker will know what to do’ as important enabling factors (Table 7).* |
| Discussion: |  |
| 29. This might be a place where my comments about the intention of screening always being disclosure could be addressed. | As stated above, we have responded to this important point by adding to the Discussion on page 24:  *Women’s responses in the study also point to the benefits of screening that extend beyond disclosure of IPV, with participants commenting on the value of creating opportunities for women to get help, education and awareness raising about IPV, affirming women’s rights to safety and empowering women who may feel isolated.* |
| 30. While I do not want to put undue weight on the responses of people who disapproved or would be uncomfortable with IPV screening, is it possible for the authors to provide a little interpretation about the reasons those few people gave against screening, and what that might suggest about the approach that refugee service providers take? I realize these were very few in number compared to those who support this practice, but providers often say that fear of offending patients (particularly related to cultural relativism) is one reason why they don’t want to talk about IPV, so acknowledging that while some participants felt uncomfortable with being asked, it was far from the majority. We need to move away from stereotypes about the overall acceptability of IPV among some communities. | This point is now addressed with the addition of the following on page 23:  *While the great majority of participants were in agreement with being asked the screening questions, 14% were not in favour with responses indicating concerns about intrusion into family privacy, discomfort with the questions, concern about confidentiality and believing the questions were not necessary. It suggests that additional explanation may be needed from settlement service caseworkers to ensure women are informed about the context and rationale for screening, and have opportunity to discuss any concerns.* |
| Limitations: |  |
| 31. I do not understand how service improvement by SETS to better respond to clients experiencing IPV was a limitation of this study, looking at the acceptability of IPV screening in the setting. In terms of the research team offering assistance to the SETS sites, it would be useful to reflect on what that assistance was (presumably IPV training, support with asking difficult questions, etc.) and rather than seeing that as a limitation, reflect on it as a necessary part of a screening program for other refugee assistance settings. | Agreed. This has been deleted from the Limitations section, rephrased and added to the Implications section:  *The successful implementation of the intervention in this study was assisted by the active role of the research team in providing pre-intervention training, regular debriefing opportunities with implementing staff, refresher training and study tools. Training, ongoing support, organisational mandate, referral protocols and user-friendly tools in community languages are required for effective implementation of IPV screening and response in settlement services.* |
| 32. Is an additional limitation that some participants in the survey did not actually receive the intervention, but cannot be separated from those who just don’t remember if they received the intervention? That seems worth reflecting on. | We don’t think this is a major limitation. Those who don’t remember being asked and those who weren’t asked both gave survey responses without reference to an experience of the intervention at the service. The survey questions were the same for these two groups. |
| Tables |  |
| 33. I will let the copy editors work with authors on formatting the tables. I don’t usually see shaded lines, and while I figured out what “IPV+ve” and “IPV-ve” mean, I don’t think it’s standard. It’s also nice when I see information about the population included in the title, even just something as simple as “all participants.” | ‘IPV+ve’ and ‘IPV-ve’ have been replaced in Tables 6 and 7. We are happy to change the table formatting in line with any journal specifications. |
